# Using the H2O Automatic Machine Learning Algorithms to Identify Predictors of Web-Based Medical Record Nonuse Among Patients in a Data-Rich Environment: Mixed Methods Study

**DOI:** 10.2196/41576

**Published:** 2023-06-19

**Authors:** Yang Chen, Xuejiao Liu, Lei Gao, Miao Zhu, Ben-Chang Shia, Mingchih Chen, Linglong Ye, Lei Qin

**Affiliations:** 1 School of Statistics, University of International Business and Economics Beijing China; 2 School of Law, University of International Business and Economics Beijing China; 3 School of Statistics, Huaqiao University Xiamen China; 4 Graduate Institute of Business Administration, College of Management, Fu Jen Catholic University New Taipei City Taiwan; 5 Artificial Intelligence Development Center, Fu Jen Catholic University New Taipei City Taiwan; 6 School of Public Affairs, Xiamen University Xiamen China; 7 Dong Fureng Institute of Economic and Social Development, Wuhan University Wuhan China

**Keywords:** web-based medical record, predictors, H2O’s automatic machine learning, Health Information National Trends Survey, HINTS, mobile phone

## Abstract

**Background:**

With the advent of electronic storage of medical records and the internet, patients can access web-based medical records. This has facilitated doctor-patient communication and built trust between them. However, many patients avoid using web-based medical records despite their greater availability and readability.

**Objective:**

On the basis of demographic and individual behavioral characteristics, this study explores the predictors of web-based medical record nonuse among patients.

**Methods:**

Data were collected from the National Cancer Institute 2019 to 2020 Health Information National Trends Survey. First, based on the data-rich environment, the chi-square test (categorical variables) and 2-tailed *t* tests (continuous variables) were performed on the response variables and the variables in the questionnaire. According to the test results, the variables were initially screened, and those that passed the test were selected for subsequent analysis. Second, participants were excluded from the study if any of the initially screened variables were missing. Third, the data obtained were modeled using 5 machine learning algorithms, namely, logistic regression, automatic generalized linear model, automatic random forest, automatic deep neural network, and automatic gradient boosting machine, to identify and investigate factors affecting web-based medical record nonuse. The aforementioned automatic machine learning algorithms were based on the R interface (R Foundation for Statistical Computing) of the *H2O* (H2O.ai) scalable machine learning platform. Finally, 5-fold cross-validation was adopted for 80% of the data set, which was used as the training data to determine hyperparameters of 5 algorithms, and 20% of the data set was used as the test data for model comparison.

**Results:**

Among the 9072 respondents, 5409 (59.62%) had no experience using web-based medical records. Using the 5 algorithms, 29 variables were identified as crucial predictors of nonuse of web-based medical records. These 29 variables comprised 6 (21%) sociodemographic variables (age, BMI, race, marital status, education, and income) and 23 (79%) variables related to individual lifestyles and behavioral habits (such as electronic and internet use, individuals’ health status and their level of health concern, etc). H2O’s automatic machine learning methods have a high model accuracy. On the basis of the performance of the validation data set, the optimal model was the automatic random forest with the highest area under the curve in the validation set (88.52%) and the test set (82.87%).

**Conclusions:**

When monitoring web-based medical record use trends, research should focus on social factors such as age, education, BMI, and marital status, as well as personal lifestyle and behavioral habits, including smoking, use of electronic devices and the internet, patients’ personal health status, and their level of health concern. The use of electronic medical records can be targeted to specific patient groups, allowing more people to benefit from their usefulness.

## Introduction

### Background

Regular review of self–medical records by patients can enhance patient-doctor communication and facilitate disease treatment. Effective communication can increase patient satisfaction, acceptance, adherence, and co-operation with the medical team. It can also improve a patient’s physiological and functional status [1]. Conversely, poor communication between doctors and patients can lead to poor quality and continuity of care [2]. Therefore, ensuring good communication by recording, processing, and sharing health information with patients is a necessary and integral part of the health care process. Encouraging patients to use medical records can reduce unnecessary duplication of testing and treatment [3].

Before the advent of electronic medical records, traditional paper-based medical records written in technical language and comprising raw data were provided to health care professionals. However, such medical records can be worrying and confusing for patients. Consequently, clinical trials that provided written records to patients at the time reported that the use of medical records by patients had little success in enhancing communication and facilitating disease treatment [4-7]. However, with the advent of electronic storage of medical records and the internet, patients can be provided with web-based access to their medical records. Internet-accessible medical records may be particularly helpful to patients compared with centrally stored paper-based medical records. Patients can review web-based medical records repeatedly at their convenience. The readability optimization of web-based cases and the increasing popularity of internet medical information have made understanding web-based medical records easier for patients. Moreover, with the current COVID-19 pandemic, the use of web-based medical records may become more prevalent.

Studies have shown that providing patients with internet-accessible medical records may lead to modest benefits. For example, overall adherence to medical advice improved among patients using web-based medical records. A trend of improvement in satisfaction with doctor-patient communication has also been observed [8]. However, many patients avoid using web-based medical records despite their greater availability and readability. Historically, this was possibly because patients had little access to web-based medical records. For example, in 2013, only 3 in 10 patients gained access to medical records, and almost half of those who gained access viewed their web-based records at least once [9]. What factors influence the use of web-based medical records in the current population? This study explored the factors that influence people’s nonuse of web-based medical records.

Some studies have applied traditional statistical methods to explore the relationship between certain factors and web-based medical record nonuse. For example, using univariable and multivariable regression models, Gerber et al [10] analyzed the use of MyChart (a personal health record portal for electronic medical record systems) among patients attending a National Cancer Institute–designated cancer center and predictors of MyChart use. Using data from the Health Information National Trends Survey (HINTS) cycle 3, Elkefi et al [9] applied descriptive statistics and chi-square tests to explore why patients tended to avoid using web-based medical records and compared patients’ perceptions of web-based medical records based on demographics and cancer diagnoses. On the basis of 2017 to 2018 HINTS data, Patel and Johnson [11] used descriptive statistics and hypothesis testing to assess individuals’ access, viewing, and use of their web-based medical records and the use of smartphone health apps and other electronic devices in 2017 and 2018. Trivedi et al [12] used multivariable logistic regression (LR) analyses to examine the association between sociodemographic and health care–related factors on being offered access to web-based medical records and accessing web-based medical records and cited reasons for not accessing web-based medical records. These studies used traditional and relatively simple statistical methods, and the selection of predictors has certain limitations. As in the research by Elkefi et al [9], predictors relate only to demographic variables and cancer diagnoses. Screening of predictors based on a data-rich environment can be optimized.

With the rapid development of artificial intelligence, machine learning methods have received increasing attention. Machine learning algorithms are used in a wide variety of applications, such as in medicine and health care, where it is difficult or unfeasible to develop conventional algorithms for necessary tasks [13]. Compared with traditional regression-based statistical methods, machine learning is data-driven and has the advantage of not assuming the distribution and relationship of predictors, and machine learning algorithms are good at handling data that are multidimensional and multivariety. Deep learning is a step forward, which makes feature engineering part of the learning task, reducing the algorithm’s dependence on feature engineering. However, the parameters of the machine learning method greatly influence model accuracy. Incorrect parameter selection and a small sample size can both lead to reduced model performance. Some parameters (such as the number of trees, learning rate, and number of leaf nodes in the random forest method) determine the structure and training method of the model, which affects prediction performance. To take full advantage of the relevant machine learning algorithms, an appropriate strategy must be developed to determine the parameters.

### Objectives

In this study, explanatory variables were chosen based on a data-rich environment. Data for this study were collected from the National Cancer Institute 2019 to 2020 HINTS. The HINTS regularly collects nationally representative data about the American public’s knowledge of, attitudes toward, and use of cancer- and health-related information; therefore, this study is based on the relevant background in the United States. We used almost all the questions in the questionnaire as possible predictors, thus avoiding the subjectivity of manual screening. To resolve the parameter selection problem of machine learning algorithms, this study adopted the current popular *H2O* (H2O.ai) automatic machine learning algorithms to realize the automation of the entire process, from construction to the application of the machine learning model. At the same time, we also used the traditional statistical method of LR. To the best of our knowledge, this is the first study that applied a range of H2O’s automatic machine learning algorithms to such a large representative sample based on a data-rich environment. We implemented a combination of H2O’s automatic machine learning methods and a data-rich environment. Predictors of web-based medical record nonuse were identified based on the results of the H2O automatic machine learning methods.

## Methods

### Data Source

Data for this study were collected from the National Cancer Institute 2019 to 2020 HINTS. The HINTS regularly collects nationally representative data about the American public’s knowledge of, attitudes toward, and use of cancer- and health-related information. Survey researchers use the data to understand how adults (aged ≥18 years) use different communication channels, including the internet, to obtain vital health information for themselves and their loved ones. This study analyzed merged data from cycles 3 to 4. Data from cycle 3 were collected between January 2019 and May 2019, and those from cycle 4 were collected from February 2019 to June 2019. We screened the respondents based on the target-dependent variable (ie, web-based medical record nonuse), leaving respondents with no missing values in the target-dependent variable. Finally, 9072 respondents were screened.

### Ethical Considerations

The HINTS administration was approved by the institutional review board at Westat Inc and deemed exempt by the National Institutes of Health Office of Human Subjects Research. This exemption also extends to this study. HINTS data are available for public use. Additional information on the survey design is available on the HINTS website.

### Statistical Analysis

Explanatory variables were selected in this study based on a data-rich environment, and all questions in the questionnaire that could be answered by all participants were selected (*P*_0_=141; variables that could only be answered by a specific group were not considered, such as questions only for females, eg, whether they had been screened for cervical cancer). The sociodemographic characteristics and other relevant variables of individuals who had or had not used web-based medical records were compared using chi-square tests for categorical variables and 2-tailed *t* tests for continuous variables. According to the results of the aforementioned statistical tests, significant variables were selected. Preliminary screening of variables was completed (*P*_1_=49; some variables were merged and answers were regrouped; refer to Multimedia Appendix 1 for details). Samples with missing values for the preliminary screened variables were excluded, and accordingly, a total of 4827 samples were obtained. On the basis of these samples, 5 algorithms were used for modeling: LR, automatic generalized linear model (auto-GLM), automatic random forest, automatic deep neural network (auto–deep learning), and automatic gradient boosting machine (auto-GBM). Of them, LR is a traditional statistical method, and the last 4 are automated machine learning algorithms based on the R interface (R Foundation for Statistical Computing) of the H2O extensible machine learning platform.

We divided the data set as follows: 80% of the data were used as the training set, and 20% of the data were used as the test set. We used the method of 5-fold cross-validation on the training data to determine hyperparameters and used the selected optimal hyperparameters to fit the model using all training data and make predictions on the test set. To evaluate the predictive accuracy of the models, we reported the accuracy, precision, recall, *F*_1_-score, and area under the curve (AUC) of the validation set (validation set results for 5-fold cross-validation in the training set) and test set. The LR model selected predictors through backward selection and stepwise regression. The relative effects of the predictors in the LR model were measured based on crude odds ratios (ORs), whereas the variability and significance were assessed based on CIs and the corresponding P values. Variable importance values were used in the other 4 H2O automatic machine learning classification algorithms to identify predictors (variables with higher importance indexes were screened as predictors, and a 5-fold cross-validation method was used to select important variables by using all data). All statistical analyses were performed using the R software (version 4.1.2). In this study, *P*<.05 was considered statistically significant.

### Measures

#### Nonuse of Web-Based Medical Records

Web-based medical records are used to organize processes in clinical and outpatient settings and forge doctor-patient communication that establishes mutual understanding and trust. The variable “nonuse of web-based medical records” in this study was calculated based on the following question in the HINTS: “How many times did you access your web-based medical record in the last 12 months?” We used this question to identify users and nonusers of web-based medical records. The respondents who reported accessing their web-based medical records at least once were coded as users, and those who reported accessing their records 0 times were coded as nonusers.

#### Demographic and Other Related Variables

Demographic variables of interest (dichotomized for analysis) included sex (male and female), race and ethnicity (non-Hispanic White and racial and ethnic minority group), education (high school or lower and more than high school), income ranges (<US $20,000 and ≥US $20,000), area (nonmetropolitan and metropolitan), and marital status (married and not married), as well as numerical demographic variables, including age (continuous years) and BMI.

For further analysis, we selected as many variables as possible from the HINTS database to identify their relationship with the use of web-based medical records. Statistical tests were performed on almost all variables in the questionnaire, including chi-square tests for categorical variables and 2-tailed *t* tests for continuous variables. The variables that passed the significance test were used as potential predictors, as follows (consistent with the question blocks in the questionnaire): 6 variables, such as *Confidence in access to health information*, in part A (looking for health information); 6 variables, such as *Internet use*, in part B (using the internet to find information); 2 variables, such as *Have regular health providers*, in part C (your health care); 3 variables, such as *Health provider maintain MR (medical record)*, in part D (medical records); *Care for someone* in part E (caregiving); 7 variables, such as *General health*, in part F (your overall health); 2 variables, such as *Notice calorie information*, in part G (health and nutrition); 2 variables, such as *Exercise days per week*, in part H (physical activity and exercise); 3 variables, such as *Smoke*, in part K (tobacco products; this part of the questionnaire was about the respondents’ consumption of tobacco products); 3 variables, such as *Ever tested colon cancer*, in part L (cancer screening and awareness); *Ever had cancer* in part M (your cancer history); 4 variables, such as *Everything cause cancer*, in part N (beliefs about cancer); and 1 numerical variable, *Sitting time per day*.

Specific variables and their descriptive statistics are shown in Multimedia Appendix 2, and Multimedia Appendix 1 lists details of some of the aforementioned variables, including demographic variables and variables adjusted for research needs with the readjustment information.

### Machine Learning Methods

#### LR Model

LR is a generalized linear regression analysis model that is part of supervised learning in machine learning. LR usually uses numerical or categorical independent variables *x*_1_, *x*_2_,...,*x*_n_ to predict the value of the categorical dependent variable *y* to determine the probability that *y* belongs to a particular category [14].

*p*(*y* = 1 | *x*_1_, *x*_2_,...,*x*_n_) = (*e^β^*^_0_ +^
*^β^*^_1_^*^x^*^_1_ + ... +^
*^β^*^_n_^*^x^*^_n_^)/(1 + *e^β^*^_0_ +^
*^β^*^_1_^*^x^*^_1_ + ... +^
*^β^*^_n_^*^x^*^_n_^) **(1)**

The OR expresses the ratio between the probability *p* that the dependent variable *y* is 1 and the probability 1 − *p* that the dependent variable *y* is 0. The OR is related to the interpretability of LR. When *x*_i_ is increased by 1, the odds become the original *e*^βi^ times.

*logit*(*p*) = ln(*p*/1−*p*) = *β*_0_ + *β*_1_*x*_1_ + ... + *β*_n_*x*_n_
**(2)**

In the aforementioned formula, β_1_, β_2_,..., β_n_ are the coefficients that measure the contribution of the independent variables *x*_1_, *x*_2_,...,*x*_n_ to *y*. If the coefficient β is positive, *e*^β^>1 and the factor have a direct correlation with *y*, whereas if β is negative, *e*^β^ is between 0 and 1.

#### H2O’s Auto-GLM

Generalized linear models (GLMs) were proposed and published by Nelder and Wedderburn [15] in 1972. It is a modeling method that can solve the problem that ordinary linear regression models cannot handle discrete dependent variables. GLM is an extension of the linear model and establishes the relationship between the mathematical expectation of the response variable and the linear combination of predictor variables through a link function. In this study, 5-fold cross-validation was adopted on the data set to select the hyperparameters of the model, and the selection range of the regularization parameter was (0, 1). Ridge regression (α=0) was used in the regression of the GLM for the variable selection and final classification. The importance of the variable was judged according to the “absolute value of the normalization coefficient” indicator; the larger the value, the greater the importance of the variable. This study used the “h2o.glm” function in the “h2o” package to build a GLM for the classification of web-based medical record use.

H2O is an open-source, in-memory, distributed, fast, and scalable machine learning and predictive analytics platform that allows users to build machine learning models on data and avoid the tedious process of manual hyperparameter tuning. H2O supports traditional (or “Cartesian”) grid searches. In a Cartesian grid search, users specify a set of values for each hyperparameter that they want to search, and H2O trains a model for every combination of the hyperparameter values. This means that, if we have 3 hyperparameters and specify 5, 10, and 2 values for each, the grid will contain a total of 5 × 10 × 2 = 100 models. After the grid search is complete, the user can query the grid object and sort the models by a specific performance metric (eg, “AUC”) and select the locally best model within the specified parameter range.

#### H2O’s Automatic Random Forest

The random forest is a multivariate statistical technique that considers an ensemble (forest) of trees for efficiency and predictive power [16]. Random forest uses a bagging technique (bootstrap aggregation) to select resamples randomly and choose a random sample of variables at each tree node as the training data set for model calibration. As the random selection of the training data set may affect the model’s results, a large set of trees is applied to guarantee model stability. In this study, the selection range of the number of trees was between 100 and 500, the selection range of the number of variables in the variable selection set at the node of the tree was approximately *p*^0.5^ (*p* is the total number of variables), and the maximum tree depth was selected from 10 to 30. When selecting variables, the parameters of the final model selected under 5-fold cross-validation were as follows: the number of trees was 150, the number of variables contained in the variable selection set at the node of the tree was 7, and the maximum depth of the tree was 10. The importance of the variable was judged according to the “mean decrease gini” indicator, where the larger the value, the greater the importance of the variable. When fitting the model, the final parameters were as follows: the number of trees was 300, the number of variables included in the variable selection set at the node of the tree was 2, and the maximum depth of the tree was 10. Model-fitting processes were implemented using the “h2o.randomForest” function in the “h2o” package. Parameter tuning was implemented using the “h2o.grid” function in the “h2o” package by grid searching for parameters.

#### H2O’s Auto–Deep Learning

The concept of deep learning originates from the study of artificial neural networks, and a multilayer perceptron with multiple hidden layers is a basic deep learning structure. Deep learning algorithms try to identify potential relationships in a data set by mimicking human brain functions. Similar to the human brain structure, deep learning models consist of neurons in complex and nonlinear forms. Deep learning models have 3 basic types of layers: input, hidden, and output layers. Each neuron in the current layer is connected to the input signal of each neuron in the previous layer. In each connection process, the signal from the previous layer is multiplied by a weight, and a bias is added and then passed through a nonlinear activation function through multiple composites of simple nonlinear functions to achieve a complex input space–to–output space map. In this study, the input values were observations of 49 variables, and the output value was the probability of the use of web-based medical records. When training the model, the number of hidden layers was 2 to 3; the number of nodes in the first layer was between 100 and 200; the number of nodes in the second layer was between 50 and 100; and the number of nodes in the third layer was 5, if any. The activation function was selected from the rectifier and rectifier with dropout; dropout ratio defaults to 0.5. The deep learning model chosen using 5-fold cross-validation to be applied for selecting variables contained 3 hidden layers, each with 100, 50, and 5 nodes. When training the model, a 50% random dropout of the nodes was set to prevent overfitting. The variable importance of the model was measured using the combination of absolute values of the coefficients. The final model for classification contained 3 hidden layers, each with 200, 50, and 5 nodes with 50% random dropout, which provided the highest mean AUC in the test set of the model in 5-fold cross-validation. In this study, we used the “h2o.deeplearning” function in the “h2o” package to realize the deep learning algorithm.

#### H2O’s Auto-GBM

The gradient boosting machine (GBM) algorithm is a type of boosting algorithm. GBM is a model that trains decision trees sequentially. Each decision tree is based on the errors of the previous tree. The core idea is to generate various weak learners in series, and the goal of each weak learner is to fit the negative gradient of the loss function of the previous accumulated model. After adding the weak learner, it enables the accumulated model loss to decrease along the negative gradient direction. It uses different weights to linearly combine the basic learners to ensure that learners with greater performance can obtain larger weights. The most commonly used base learners are tree models. Variable importance is determined by calculating the relative influence of each variable: whether that variable is selected to split during the tree-building process and how much the squared error improves or decreases as a result. We used 5-fold cross-validation in both the variable selection and classification model-fitting procedures, which would provide indicators for the selection of optimal hyperparameters. In the process of using a grid search for hyperparameter optimization, it is necessary to train the models under different hyperparameter specifications and evaluate the goodness of fit of the models under these specifications through 5-fold cross-validation. The number of trees ranged from 100 to 500, and the final parameter was set to 300 in both the variable selection and classification model-fitting procedures, which promises a balance in the training and test set errors in 5-fold cross-validation. In addition, the selection of the learning rate ranged from 0.01 to 0.10, and the final parameter was set to 0.01. The maximum depth was between 10 and 30, and the final parameter was set to 10 in both the variable selection and classification model-fitting procedures, which implements a trade-off between model bias and model variance. This study used the “h2o.gbm” function in the “h2o” package to build a GBM for the use of web-based medical record classification problems. Parameter tuning was implemented using the “h2o.grid” function in the “h2o” package by grid searching for parameters.

## Results

### Descriptive Statistics

The merged data sets from HINTS cycles 3 and 4 yielded a sample of 9072 respondents, including 5409 (59.62%) nonusers and 3663 (40.38%) users of web-based medical records. Multimedia Appendix 2 presents the frequencies and proportions of the variables. The chi-square test of categorical variables and the 2-tailed *t* test of continuous variables showed significant differences in some variables between nonusers and users of web-based medical records (*P*<.05). Among the categorical variables, respondents who chose the following options comprised a significantly higher proportion (*P*<.05) of the group not using web-based medical records: “male,” “trust information about health or medical topics from religious organizations and leaders,” “have no drink,” and “smoke more.” For example, in this group, male individuals accounted for 45.23% (2196/4855) of the respondents, whereas in the group using web-based medical records, the percentage decreased to 38.65% (1331/3444). The same was true for the other aforementioned variables: “trust information about health or medical topics from religious organizations and leaders” (1501/4934, 30.42% vs 824/3573, 23.06%), “have no drink” (2623/4692, 55.9% vs 1575/3421, 46.04%), and “smoke more” (824/3573, 39.38% vs 1264/3630, 34.82%). However, those choosing “Non-Hispanic White” (3492/4885, 71.48% vs 2686/3488, 77.01%), “have higher education level” (3471/5212, 66.6% vs 3160/3598, 87.83%), “have higher income level” (3569/4741, 75.28% vs 3042/3349, 90.83%), “in marriage” (2481/5198, 47.73% vs 2252/3600, 62.56%), “ever looked for information about cancer” (2378/5344, 44.50% vs 2429/3646, 66.62%), “use Internet” (3841/5379, 71.41% vs 3501/3650, 95.92%), “use electronic” (3752/5346, 70.18% vs 3572/3642, 98.08%), “use Internet for health use” (3337/5289, 63.09% vs 3111/3636, 85.56%), “have regular healthcare provider” (3285/5293, 62.06% vs 2930/3625, 80.83%), “caring someone” (723/5216, 13.86% vs 656 /3604, 18.20%), “general health relative good” (4343/5331, 81.47% vs 3169/3625, 87.42%), “high confidence in ability to take good care of own health” (5036/5337, 94.36% vs 3503/3632, 96.45%), and others were significantly higher (*P*<.05) in the group using web-based medical records. Among the numeric variables, mean values of age were significantly higher (*P*<.05) in the group not using web-based medical records, whereas time spent sitting was significantly higher (*P*<.05) in the group using web-based medical records. The 2-tailed *t* test of continuous variables also showed no significant difference (*P*>.05) in some variables between individuals who had and had not used web-based medical records. In other words, the proportions of these variables were similar between the 2 groups. As for BMI, the average value in both groups was approximately 28.5 (SD 0.1).

### Machine Learning Model Results

As shown in Tables 1 and Table 2, a total of 29 predictors of nonuse of web-based medical records variables (*Age, Sitting time per day, BMI, Confidence in access to health information, education, Electronic means use, Ever tested colon cancer, Everything cause cancer, Number of visits to health provider, Have electronic device, income, Obesity affects cancer onset, Social media use, Little interest, Marital status, Offered access to MR by health provider, Offered access to MR by health insurer, Health provider maintain MR, race, Have regular health providers, Seek cancer information, Shared health information, Smoke, Exercise days per week, Strength training days per week, Trust doctor, Trust religious organizations, Internet use, and Electronic wearable device use*) were selected in all the 5 algorithms, and 7 variables (*Age, Electronic means use, Number of visits to health provider, Offered access to MR by health provider, Offered access to MR by health insurer, Health provider maintain MR, and Internet use*) were selected simultaneously in the 5 algorithms.

**Table 1:** Predictors of nonuse of web-based medical records (MRs) using the logistic regression algorithm.

Predictor			OR^a^ (95% CI)
Race (reference: non-Hispanic White)			1.04 (0.90-1.19)
Education (reference: high school or lower)			0.32 (0.27-0.37)
Income (reference: <US $20,000)			0.35 (0.29-0.43)
Marital status (reference: not married)			0.60 (0.54-0.68)
Trust doctor^b^ (reference: low_level)			0.73 (0.53-0.99)
Trust religious organization^b^ (reference: low_level)			1.31 (1.15-1.50)
Internet use (reference: no)			0.12 (0.09-0.16)
Electronic means use (reference: no)			0.05 (0.03-0.07)
Electronic wearable device use (reference: no)			0.40 (0.35-0.45)
**Shared health information (reference: N/A^c^)**			
	No	0.71 (0.58-0.87)	
	Yes	0.26 (0.20-0.33)	
Social media use (reference: no)			0.37 (0.32-0.43)
Have regular health providers^d^ (reference: no)			0.36 (0.32-0.42)
**Number of visits to health provider^e^ (reference: none)**			
	1 time	0.24 (0.18-0.33)	
	2 times	0.19 (0.15-0.26)	
	3 times	0.15 (0.12-0.21)	
	4 times	0.14 (0.11-0.19)	
	5-9 times	0.10 (0.08-0.14)	
	≥10 times	0.10 (0.08-0.14)	
**Health provider maintain MR (reference: no)**			
	Yes	0.11 (0.06-0.22)	
	Don’t know	1.14 (0.56-2.30)	
**Offered access to MR by health provider^f^ (reference: no)**			
	Yes	0.03 (0.03-0.04)	
	Don’t know	0.83 (0.57-1.22)	
**Offered access to MR by health insurer^f^ (reference: no)**			
	Yes	0.21 (0.19-0.25)	
	Don’t know	0.82 (0.71-0.95)	
**Strength training days per week (reference: none)**			
	1-3 days per week	0.63 (0.56-0.71)	
	4-7 days per week	0.84 (0.70-1.01)	
Ever tested colon cancer (reference: no)			0.74 (0.66-0.83)
Age			1.01 (1.00-1.01)
BMI			0.99 (0.99-1.00)

^a^OR: odds ratio.

^b^In general, how much would you trust information about cancer from a doctor/government health agencies/charitable organizations/religious organizations and leaders? (Supplement to the variable-related questions in the survey).

^c^N/A: not applicable.

^d^Not including psychiatrists and other mental health professionals, is there a particular doctor, nurse, or other health professional that you see most often? (Supplement to the variable-related questions in the survey).

^e^In the past 12 months, not counting times you went to an emergency room, how many times did you go to a doctor, nurse, or other health professional to get care for yourself? (Supplement to the variable-related questions in the survey).

^f^Have you ever been offered online access to your medical records by your health care provider/health insurer? (Supplement to the variable-related questions in the survey).

Table 1 shows significant predictors of nonuse of web-based medical records in LR (*P*<.05). The variables in LR were screened using 2 methods: backward selection and stepwise regression. The results obtained using the 2 variable selection methods were consistent, and 20 significant variables were finally selected.

The results of LR showed that sociodemographic indicators, such as age, BMI, education, marital status, income, and race, significantly affected the nonuse of web-based medical records, whereas sex and area had no significant effect on the prediction of nonuse of web-based medical records. On the basis of sociodemographic variables, people who were relatively older (OR 1.01, 95% CI 1.00-1.01), had a relatively lower BMI (OR 0.99, 95% CI 0.99-1.00), had relatively lower education (higher education OR 0.32, 95% CI 0.27-0.37), were not married (married OR 0.60, 95% CI 0.54-0.68), had a lower income (higher income OR 0.35, 95% CI 0.29-0.43), and belonged to racial and ethnic minority groups (OR 1.04, 95% CI 0.9-1.19) were more likely to not use web-based medical records. People who did not often access the internet or send and receive emails (OR 0.12, 95% CI 0.09-0.16), had not used a computer or smartphone to inquire about medical information in the past 12 months (OR 0.05, 95% CI 0.03-0.07), did not often use a wearable device to track health (OR 0.40, 95% CI 0.35-0.45), did not share health information from an electronic monitoring device or smartphone with a health professional in the previous 12 months, and people who had not used social media in the last 12 months (OR 0.37, 95% CI 0.32-0.43) were more inclined not to use web-based medical records. Moreover, those who were less concerned about their own health were more likely to not use web-based medical records. People who were more likely to not use web-based medical records tended to be those who did not see a particular doctor or health care professional frequently (OR 0.36, 95% CI 0.32-0.42); had not gone to a doctor, nurse, or other health professional to receive care in the last 12 months; did not have doctors or other health care providers maintain their medical records in a computerized system (OR 0.11, 95% CI 0.06-0.22); were not offered web-based access to their medical records by the health care provider (OR 0.03, 95% CI 0.03-0.04); were not offered web-based access to their medical records by the health insurer (OR 0.21, 95% CI 0.19-0.25); and did not perform leisure-time physical activities specifically designed to strengthen muscles. People who strongly trusted information about health or medical topics from religious organizations and leaders (OR 1.31, 95% CI 1.15-1.50), trusted information about health or medical topics from a doctor only a little (OR 0.73, 95% CI 0.53-0.99), and did not check for colon cancer (OR 0.74, 95% CI 0.66-0.83) were more inclined to not use web-based medical records.

**Table 2:** Predictors of web-based medical record (MR) nonuse built using automatic generalized linear model (auto-GLM), automatic random forest, auto–deep learning, and automatic gradient boosting machine (auto-GBM).

Model and predictor		Importance scores
**Auto-GLM**		
	Offered access to MR by health provider^a^	100
	Electronic means use	47.41
	Health provider maintain MR	22.72
	Number of visits to health provider^b^	21.63
	Age	17.11
	Offered access to MR by health insurer^a^	14.94
	Electronic wearable device use	13.91
	Have regular health providers^c^	13.10
	Shared health information	12.06
	Internet use	10.97
	Ever tested colon cancer	9.64
	Social media use	9.30
	Income	6.45
	Education	6.27
	Race	6.23
**Automatic random forest**		
	Offered access to MR by health provider	100
	Electronic means use	19.15
	Offered access to MR by health insurer	19.02
	Health provider maintain MR	16.03
	Number of visits to health provider	10.20
	Internet use	6.88
	Have regular health providers	5.97
	Age	5.43
	Shared health information	4.71
	Electronic wearable device use	4.58
	Sitting time per day	4.29
	BMI	4.15
	Have electronic device	4.13
	Education	2.96
	Sitting time per day	2.83
**Auto–deep learning**		
	Offered access to MR by health provider	100
	Electronic means use	51.18
	Health provider maintain MR	40.19
	Internet use	34.94
	Offered access to MR by health insurer	34.41
	Number of visits to health provider	33.24
	Little interest^d^	33.02
	Social media use	32.59
	Smoke	32.22
	Confidence in access to health information	31.86
	Race	31.80
	Sitting time per day	31.71
	Age	31.62
	Obesity affects cancer onset	30.91
	Have electronic device	30.78
**Auto-GBM**		
	Offered access to MR by health provider	100
	Electronic means use	15.87
	Number of visits to health provider	6.84
	Age	3.97
	Offered access to MR by health insurer	3.95
	Sitting time per day	3.08
	Electronic wearable device use	2.48
	Shared health information	2.08
	Internet use	1.80
	BMI	1.49
	Health provider maintain medical records	1.46
	Have electronic device	1.28
	Have regular health providers	1.23
	Exercise days per week	1.13
	Everything cause cancer	0.97

^a^Have you ever been offered online access to your medical records by your health care provider/health insurer? (Supplement to the variable-related questions in the survey).

^b^In the past 12 months, not counting times you went to an emergency room, how many times did you go to a doctor, nurse, or other health professional to get care for yourself? (Supplement to the variable-related questions in the survey).

^c^Not including psychiatrists and other mental health professionals, is there a particular doctor, nurse, or other health professional that you see most often? (Supplement to the variable-related questions in the survey).

^d^Over the past 2 weeks, how often have you been bothered by little interest or pleasure in doing things? (Supplement to the variable-related questions in the survey).

The essence of this study is a binary classification problem of judging whether individuals have used web-based medical records based on a set of inputs, such as education and income. Therefore, we evaluated the 5 methods using a series of evaluation metrics commonly used for classification algorithms. Table 3 presents the accuracy, precision, recall, *F*_1_-score, and AUC values for the 5 machine learning methods on the validation and test sets. Accuracy is a metric of a classification model that measures the percentage of correct classification accounts for the total number of classifications. Precision is the proportion of correctly predicted positives to all predicted positives, whereas recall is the proportion of correctly predicted positives to all actual positives. The *F*_1_-score is the harmonic mean of precision and recall. From the results of the verification set, LR had 3 indicators that performed best, with an accuracy of 83.35%, a recall of 88.97%, and an *F*_1_-score of 83.59%. However, the performance of LR on the test set was inferior to that of machine learning methods. In the test set, auto-GLM had the highest accuracy (82.49%), auto-GBM had the highest precision (79.73%), and automatic random forest had the highest recall (87.15%) and AUC (82.87%). AUC is not affected by the classification threshold and data distribution and, thus, reflects the overall classification power of the model. Automatic random forest had the highest AUC in both the validation (88.52%) and test (82.87%) sets. Therefore, in general, we were more inclined to choose the automatic random forest as the optimal model for predicting web-based medical record nonuse. Equations 3 to 6 provide the evaluation formulas for the machine learning models.


*Accuracy = (TP [True Positive] + TN [True Negative])/(TP + TN +FP [False Positive] + FN [False Negative])*
**(3)**

*Precision = TP/(TP + FP)*
**(4)**

*Recall = TP/(TP + FN)*
**(5)**

*F1-score = 2 × (Recall × Precision)/(Recall + Precision)*
**(6)**

**Table 3:** Correct classification metrics for each machine learning method.

Criterion	LR^a^		Auto-GLM^b^		Automatic random forest		Auto–deep learning		Auto-GBM^c^	
	Validation	Test	Validation	Test	Validation	Test	Validation	Test	Validation	Test
Accuracy, %	83.35	81.47	82.17	82.49	82.48	82.38	82.57	80.93	81.32	79.69
Precision, %	78.86	76.26	85.09	77.73	86.1	76.43	89.52	74.3	85.38	79.73
Recall, %	88.97	86.7	79.76	84.81	79.04	87.15	75.01	87.15	77.78	76.96
*F*_1_-score, %	83.59	82.01	82.34	81.11	82.3	81.44	81.57	80.22	81.31	78.32
AUC^d^, %	82.29	81.8	88.46	82.72	88.52	82.87	87.54	81.56	87.68	79.57

^a^LR: logistic regression.

^b^Auto-GLM: automatic generalized linear model.

^c^Auto-GBM: automatic gradient boosting machine.

^d^AUC: area under the curve.

## Discussion

### Principal Findings

Effective communication is key for delivering high-quality health care services [1]. Ensuring good communication by recording, processing, and sharing health information with patients is integral to the health care process. At present, the development of information and communications technology has allowed for the realization of web-based medical records, but because of some situations, web-based medical records are still not fully popular among patients. On the basis of demographic and individual behavioral characteristics, this study explored the predictors of web-based medical record nonuse.

We conducted a comprehensive assessment of the effects of individual sociodemographic characteristics, lifestyle, and behavioral habits on nonuse of web-based medical records. Although generalizing the factors that influence individuals’ nonuse of web-based medical records was difficult, based on the survey data, this study conducted in a data-rich variable selection environment demonstrated that an individual’s nonuse of web-based medical records is related to their sociodemographic characteristics, such as age, lifestyle, behavioral habits, and attention to health problems.

To date, numerous studies on the use of web-based medical records by patients have been based on surveys wherein data were gathered using a semistructured interview approach [17]. Using data from the National Cancer Institute 2019 to 2020 HINTS database, we applied the following question—“How many times did you access your web-based medical record in the last 12 months?”—to determine whether a person used web-based medical records. Our analysis showed that 59.62% (5409/9072) of the population in the survey samples had no experience using web-based medical records.

This study used 5 algorithms—LR, auto-GLM, automatic random forest, auto–deep learning, and auto-GBM—to identify and investigate factors affecting individuals’ nonuse of web-based medical records. Of them, LR is a traditional statistical method, and the latter 4 algorithms are part of H2O’s automatic parameterization methods. A total of 29 influencing variables concerning the use of web-based medical records were selected based on coefficient significance in the LR model and the variable importance indicators in the other 4 methods. Many well-established determinants were also identified as proof of concept for our analytical approach, such as sociodemographic characteristics [9]. Although nonlinear and ensemble algorithms exhibit better predictive performance than traditional parametric models, they are less interpretable [18]. Therefore, predictors determined by such algorithms should be evaluated in conjunction with relevant research evidence.

This study showed that sociodemographic indicators, such as age, BMI, race, marital status, education, and income, significantly affected the nonuse of web-based medical records, whereas sex and area did not significantly affect the prediction of the nonuse of web-based medical records. In addition, predictors involving personal lifestyle and behavioral habits, such as smoking, electronic device use, and internet use, also played essential roles in predicting the nonuse of web-based medical records. Finally, individuals’ health status and their level of health concern were also associated with the nonuse of web-based medical records. Of course, some regions or units do not provide web-based access to medical records, or these are not maintained by health care providers, which may directly limit the use of web-based medical records.

On the basis of some of the conclusions of this study, recommendations can be made to promote the widespread use of web-based medical records. The use of electronic equipment is also a factor affecting the use of web-based medical records. People who are not accustomed to using electronic devices, such as mobile phones and computers, generally do not access their web-based medical records. The reason for avoiding web-based medical records may not be the disadvantage of web-based medical records itself but the resistance to electronic products, which is more common in older adults. However, older adults are more likely to become sick, so web-based medical records for this group are also a direction that needs special attention and development, such as building an interface that is friendly to the older adult population and keeping the internet-accessible interface simple and clear. Web-based medical records are often used by people with more related health problems. A study by the Office of the National Coordinator of Health Information Technology found that individuals may not realize the value of accessing their web-based medical records until they have a medical need. Given that the patient record request process can be time consuming, it may be more beneficial to have access to a person’s data in advance of an urgent health need. Therefore, popularizing health knowledge to the public and increasing the public’s attention to health information can increase the public’s demand for health-related information to a certain extent, thereby promoting the use of web-based medical records.

This research can provide a theoretical basis for predicting individual web-based medical record use. On the basis of the predictors of people not using web-based medical records selected by machine learning algorithms, individuals who do not use such records can be identified in advance, and use of web-based medical records can be promoted among them. Thus, this would provide more effective doctor-patient communication and better health care services.

### Limitations

This study has some limitations. First, this study explored the influencing factors of nonuse of web-based medical records and discussed the correlation between each influencing factor and the target variable but did not involve the study of the influence path and influence mechanism. We considered conducting a causal analysis, but the cross-sectional survey design of the HINTS prevented us from making or testing causal claims. Second, the relationships between the selected variables were not studied further, and the screened important predictors may have certain collinearity. Third, the data were obtained using a self-report questionnaire. Therefore, we did not obtain detailed information on the nonuse of web-based medical records, and self-report bias may have affected the results. Finally, patient access to web-based medical records varies from country to country, and cultural background also has a strong impact on medical services. This study used HINTS data from the United States, and the conclusions may not be generalizable to other countries.

### Comparison With Prior Work

The use of web-based medical records can enhance patient participation and co-operation in disease treatment and enhance doctor-patient communication to promote disease treatment. This is evidenced in the study by Stewart et al [19], whose research took patients with diabetes as the research object and found that patient portals support engagement by facilitating patient access to their health information and facilitating patient-provider communication. With the advancement of internet technology and the popularization of electronic products, the use of web-based medical records has become more convenient, but its penetration rate is still not high. Therefore, it has become a research hot spot to explore the characteristics and differences between users and nonusers of web-based medical records and identify the influencing factors of low use rate.

There are many studies based on HINTS data, such as that by Anthony et al [20], who used data from the 2017 HINTS to estimate 2 separate multivariable LR models to predict the factors associated with not having been offered access and those associated with not using a portal. On the basis of the 2017 to 2018 HINTS data, Patel and Johnson [11] used descriptive statistics and hypothesis testing to assess individuals’ access, viewing, and use of their web-based medical records and the use of smartphone health apps and other electronic devices. Trivedi et al [12] used multivariable LR analyses to examine the association between sociodemographic and health care–related factors on being offered access to web-based medical records and accessing web-based medical records and cited reasons for not accessing web-based medical records. Hong et al [21] used LR to investigate the trend of patient portal use in the general population and the barriers to adoption. The aforementioned studies are all based on traditional statistical methods such as LR [12,20,21] and hypothesis testing related to association analysis [11], and the selection of influencing factors and the conclusions drawn only involve demographic variables and some variables of interest, whereas our research combines traditional statistical methods and machine learning methods to find predictors of web-based medical record use based on variable-rich environments. Furthermore, the results of machine learning methods also provide variable importance scores and rankings, which have also not been covered in previous studies. To the best of our knowledge, this is the first study to apply a range of H2O’s automatic machine learning algorithms to a nationally representative sample for optimizing the classification of web-based medical record nonuse. Compared with previous studies, we found that personal lifestyle and behavioral habits as well as individuals’ health status and their level of health concern significantly affect web-based medical record nonuse.

### Conclusions

Using data from the National Cancer Institute 2019 to 2020 HINTS database, this study applied 5 machine learning algorithms—LR (linear), auto-GLM, automatic random forest, auto–deep learning, and auto-GBM—to identify and investigate the factors that affect whether individuals use web-based medical records. Using these 5 models, 29 variables were identified as crucial predictors of nonuse of web-based medical records. When monitoring web-based medical record use trends, research should consider social factors such as age, education, BMI, and marital status, as well as personal lifestyle and behavioral habits, including smoking, use of electronic devices and the internet, patients’ personal health status, and their level of health concern. The use of electronic medical records can be targeted to specific patient groups, allowing more people to benefit from their usefulness.

The main contributions of this study are as follows: (1) using authoritative data, potential predictors were selected based on a data-rich environment, involving more comprehensive variables and avoiding unnecessary subjectivity, and (2) the key parameters of the machine learning methods considerably influenced the accuracy of the model. In this study, H2O’s automatic parameter selection methods were introduced to optimize the key parameters of the model. Compared with traditional machine learning algorithms, the H2O automatic machine learning methods effectively improved model performance.

## Appendix

Multimedia Appendix 1 Variables extracted from the Health Information National Trends Survey database for research.

Multimedia Appendix 2 Distribution of characteristics of variables in the Health Information National Trends Survey database (N=9072).

## Abbreviations

AUC: area under the curve
auto-GBM: automatic gradient boosting machine
auto-GLM: automatic generalized linear model
GBM: gradient boosting machine
GLM: generalized linear model
HINTS: Health Information National Trends Survey
LR: logistic regression
OR: odds ratio
