# Supplementary material for: Using the H2O Automatic Machine Learning Algorithms to Identify Predictors of Web-Based Medical Record Nonuse Among Patients in a Data-Rich Environment: Mixed Methods Study
Source: JMIR Med Inform. 2023 Jun 19;11:e41576. doi: 10.2196/41576 (PMC10337515; doi:10.2196/41576)
Supplement: Multimedia Appendix 2 [file medinform_v11i1e41576_app2.doc]

**Multimedia Appendix 2**

Supplementary Material Table **2.** Distribution of characteristics of variables in the Health Information National Trends Survey database (N=9072).^a^

| Variable | | | | | Nonuse of web-based medical records (n=5409) | Use of web-based medical records (n=3663) | *P* value |
| --- | --- | --- | --- | --- | --- | --- | --- |
|  | | | |  | |  |  |
| Total | | | | | 5409 (59.62) | 3663 (40.38) |  |
| **Categorical variables, n (%)** | | | | | | | |
|  | **Demographic variables** | | | | | | |
|  |  | **Sex** | | | | | <.001 |
|  |  |  | Male | | 2196 (45.23) | 1331 (38.65) |  |
|  |  |  | Female | | 2659 (54.77) | 2113 (61.35) |  |
|  |  | **Race** | | | | | <.001 |
|  |  |  | Non-Hispanic White | | 3492 (71.48) | 2686 (77.01) |  |
|  |  |  | Racial and ethnic minority group | | 1393 (28.52) | 802 (22.99) |  |
|  |  | **Education** | | | | | <.001 |
|  |  |  | More than high school | | 3471 (66.60) | 3160 (87.83) |  |
|  |  |  | High school or lower | | 1741 (33.40) | 438 (12.17) |  |
|  |  | **Income (US $)** | | | | | <.001 |
|  |  |  | ≥20,000 | | 3569 (75.28) | 3042 (90.83) |  |
|  |  |  | <20,000 | | 1172 (24.72) | 307 (9.17) |  |
|  |  | **Area** | | | | | <.001 |
|  |  |  | Metropolitan | | 4728 (87.41) | 3345 (91.32) |  |
|  |  |  | Nonmetropolitan | | 681 (12.59) | 318 (8.68) |  |
|  |  | **Marital status** | | | | | <.001 |
|  |  |  | Married | | 2481 (47.73) | 2252 (62.56) |  |
|  |  |  | Not married | | 2717 (52.27) | 1348 (37.44) |  |
|  | **Part A: looking for health information** | | | | | | |
|  |  | **Confidence in access to health information** | | | | | <.001 |
|  |  |  | High_level | | 4796 (90.78) | 3465 (95.48) |  |
|  |  |  | Low_level | | 487 (9.22) | 164 (4.52) |  |
|  |  | **Trust doctor**^b^ | | | | | <.001 |
|  |  |  | High_level | | 4932 (93.53) | 3499 (96.55) |  |
|  |  |  | Low_level | | 341 (6.47) | 125 (3.45) |  |
|  |  | **Trust government^b^** | | | | | <.001 |
|  |  |  | High_level | | 3372 (68.33) | 2695 (75.57) |  |
|  |  |  | Low_level | | 1563 (31.67) | 871 (24.43) |  |
|  |  | **Trust charitable organizations^b^** | | | | | .03 |
|  |  |  | High_level | | 2100 (43.01) | 1618 (45.42) |  |
|  |  |  | Low_level | | 2783 (56.99) | 1944 (54.58) |  |
|  |  | **Trust religious organizations^b^** | | | | | <.001 |
|  |  |  | High_level | | 1501 (30.42) | 824 (23.06) |  |
|  |  |  | Low_level | | 3433 (69.58) | 2749 (76.94) |  |
|  |  | **Seek cancer Information** | | | | | <.001 |
|  |  |  | Yes | | 2378 (44.5) | 2429 (66.62) |  |
|  |  |  | No | | 2966 (55.5) | 1217 (33.38) |  |
|  | **Part B: using the internet to find information** | | | | | | |
|  |  | **Internet use** | | | | | <.001 |
|  |  |  | Yes | | 3841 (71.41) | 3501 (95.92) |  |
|  |  |  | No | | 1538 (28.59) | 149 (4.08) |  |
|  |  | **Electronic means use** | | | | | <.001 |
|  |  |  | Yes | | 3752 (70.18) | 3572 (98.08) |  |
|  |  |  | No | | 1594 (29.82) | 70 (1.92) |  |
|  |  | **Have electronic device** | | | | | <.001 |
|  |  |  | Tablet computer | | 342 (6.50) | 114 (3.14) |  |
|  |  |  | Smartphone | | 1579 (30.01) | 954 (26.31) |  |
|  |  |  | Basic cell phone only | | 775 (14.73) | 133 (3.67) |  |
|  |  |  | None | | 384 (7.30) | 32 (0.88) |  |
|  |  |  | Multiple devices selected | | 2182 (41.47) | 2393 (66) |  |
|  |  | **Electronic wearable device use** | | | | | <.001 |
|  |  |  | Yes | | 976 (18.27) | 1360 (37.41) |  |
|  |  |  | No | | 4367 (81.73) | 2275 (62.59) |  |
|  |  | **Shared health information** | | | | | <.001 |
|  |  |  | Yes | | 593 (11.20) | 931 (25.72) |  |
|  |  |  | No | | 4076 (76.98) | 2425 (66.99) |  |
|  |  |  | N/A^m^ | | 626 (11.82) | 264 (7.29) |  |
|  |  | **Social media use** | | | | | <.001 |
|  |  |  | Yes | | 3337 (63.09) | 3111 (85.56) |  |
|  |  |  | No | | 1952 (36.91) | 525 (14.44) |  |
|  | **Part C: your health care** | | | | | | |
|  |  | **Have regular health providers^c^** | | | | | <.001 |
|  |  |  | Yes | | 3285 (62.06) | 2930 (80.83) |  |
|  |  |  | No | | 2008 (37.94) | 695 (19.17) |  |
|  |  | **Number of visits to health provider^d^** | | | | | <.001 |
|  |  |  | None | | 990 (18.51) | 153 (4.2) |  |
|  |  |  | 1 time | | 788 (14.73) | 422 (11.58) |  |
|  |  |  | 2 times | | 1031 (19.27) | 670 (18.38) |  |
|  |  |  | 3 times | | 791 (14.79) | 597 (16.38) |  |
|  |  |  | 4 times | | 686 (12.82) | 587 (16.1) |  |
|  |  |  | 5-9 times | | 648 (12.11) | 742 (20.36) |  |
|  |  |  | ≥10 times | | 415 (7.76) | 474 (13) |  |
|  | **Part D: medical records (MR)** | | | | | | |
|  |  | **Health provider maintain MR** | | | | | <.001 |
|  |  |  | Yes | | 3829 (71.29) | 3482 (95.27) |  |
|  |  |  | No | | 220 (4.1) | 24 (0.66) |  |
|  |  |  | Don’t know | | 1322 (24.61) | 149 (4.08) |  |
|  |  | **Offered access to MR by health provider^e^** | | | | | <.001 |
|  |  |  | Yes | | 1833 (34.37) | 3402 (93.1) |  |
|  |  |  | No | | 2429 (45.55) | 166 (4.54) |  |
|  |  |  | Don’t know | | 1071 (20.08) | 86 (2.35) |  |
|  |  | **Offered access to MR by health insurer^e^** | | | | | <.001 |
|  |  |  | Yes | | 715 (15.05) | 1542 (45.54) |  |
|  |  |  | No | | 2640 (55.57) | 1136 (33.55) |  |
|  |  |  | Don’t know | | 1396 (29.38) | 708 (20.91) |  |
|  | **Part E: caregiving** | | | | | | |
|  |  | **Care for someone^f^** | | | | | <.001 |
|  |  |  | Yes | | 723 (13.86) | 656 (18.2) |  |
|  |  |  | No | | 4493 (86.14) | 2948 (81.8) |  |
|  | **Part F: your overall health** | | | | | | |
|  |  | **General health** | | | | | <.001 |
|  |  |  | Relatively good | | 4343 (81.47) | 3169 (87.42) |  |
|  |  |  | Relatively bad | | 988 (18.53) | 456 (12.58) |  |
|  |  | **Confidence in taking care of yourself** | | | | | <.001 |
|  |  |  | High_level | | 5036 (94.36) | 3503 (96.45) |  |
|  |  |  | Low_level | | 301 (5.64) | 129 (3.55) |  |
|  |  | **Hearing impaired** | | | | | <.001 |
|  |  |  | Yes | | 507 (9.5) | 256 (7.06) |  |
|  |  |  | No | | 4832 (90.5) | 3372 (92.94) |  |
|  |  | **Talk about health with friends** | | | | | <.001 |
|  |  |  | Yes | | 4093 (76.68) | 3147 (86.69) |  |
|  |  |  | No | | 1245 (23.32) | 483 (13.31) |  |
|  |  | **Little interest^g^** | | | | | <.001 |
|  |  |  | Nearly every day | | 347 (6.58) | 154 (4.25) |  |
|  |  |  | More than half the days | | 379 (7.19) | 222 (6.13) |  |
|  |  |  | Several days | | 1032 (19.58) | 790 (21.82) |  |
|  |  |  | Not at all | | 3514 (66.65) | 2455 (67.80) |  |
|  |  | **Feeling hopeless^g^** | | | | | <.001 |
|  |  |  | Nearly every day | | 233 (4.44) | 104 (2.87) |  |
|  |  |  | More than half the days | | 290 (5.52) | 204 (5.63) |  |
|  |  |  | Several days | | 951 (18.11) | 714 (19.72) |  |
|  |  |  | Not at all | | 3776 (71.92) | 2599 (71.78) |  |
|  |  | **Feeling nervous^g^** | | | | | <.001 |
|  |  |  | Nearly every day | | 290 (5.51) | 156 (4.31) |  |
|  |  |  | More than half the days | | 337 (6.40) | 234 (6.46) |  |
|  |  |  | Several days | | 1186 (22.53) | 1053 (29.09) |  |
|  |  |  | Not at all | | 3451 (65.56) | 2177 (60.14) |  |
|  | **Part G: health and nutrition** | | | | | | |
|  |  | **Notice calorie information** | | | | | <.001 |
|  |  |  | Yes | | 2125 (40.07) | 2080 (57.35) |  |
|  |  |  | No | | 3178 (59.93) | 1547 (42.65) |  |
|  |  | **Drink days per week** | | | | | <.001 |
|  |  |  | None | | 2623 (55.9) | 1575 (46.04) |  |
|  |  |  | 1-3 | | 1389 (29.6) | 1277 (37.33) |  |
|  |  |  | 4-7 | | 680 (14.49) | 569 (16.63) |  |
|  | **Part H: physical activity and exercise** | | | | | | |
|  |  | **Exercise days per week** | | | | | <.001 |
|  |  |  | None | | 1661 (31.29) | 746 (20.62) |  |
|  |  |  | 1-3 days per week | | 1828 (34.43) | 1475 (40.78) |  |
|  |  |  | 4-7 days per week | | 1820 (34.28) | 1396 (38.6) |  |
|  |  | **Strength training days per week** | | | | | <.001 |
|  |  |  | None | | 3100 (58.87) | 1801 (50.07) |  |
|  |  |  | 1-3 days per week | | 1523 (28.92) | 1348 (37.48) |  |
|  |  |  | 4-7 days per week | | 643 (12.21) | 448 (12.45) |  |
|  | **Part K: tobacco products** | | | | | | |
|  |  | **Smoke** | | | | | <.001 |
|  |  |  | Yes | | 2095 (39.38) | 1264 (34.82) |  |
|  |  |  | No | | 3225 (60.62) | 2366 (65.18) |  |
|  |  | **Atitude to e-cigarette** | | | | | <.001 |
|  |  |  | Relatively less harmful | | 2194 (42.37) | 1869 (52.7) |  |
|  |  |  | Relatively more harmful | | 1000 (19.31) | 714 (20.14) |  |
|  |  |  | I don’t know | | 1984 (38.32) | 963 (27.16) |  |
|  |  | **Have Seen federal court tobacco messages^h^** | | | | | <.001 |
|  |  |  | Yes | | 2268 (42.56) | 1685 (46.38) |  |
|  |  |  | No | | 3061 (57.44) | 1948 (53.62) |  |
|  | **Part L: cancer screening and awareness** | | | | | | |
|  |  | **Ever tested colon cancer** | | | | | <.001 |
|  |  |  | Yes | | 3161 (60.67) | 2319 (64.9) |  |
|  |  |  | No | | 2049 (39.33) | 1254 (35.1) |  |
|  |  | **Ever heard hpv** | | | | | <.001 |
|  |  |  | Yes | | 3112 (58.83) | 2822 (77.87) |  |
|  |  |  | No | | 2178 (41.17) | 802 (22.13) |  |
|  |  | **Ever heard hpv vaccine** | | | | | <.001 |
|  |  |  | Yes | | 2846 (54.66) | 2667 (74.43) |  |
|  |  |  | No | | 2361 (45.34) | 916 (25.57) |  |
|  | **Part M: your cancer history** | | | | | | |
|  |  | **Ever had cancer** | | | | | <.001 |
|  |  |  | Yes | | 789 (14.9) | 648 (17.88) |  |
|  |  |  | No | | 4508 (85.1) | 2976 (82.12) |  |
|  | **Part N: beliefs about cancer** | | | | | | |
|  |  | **Everything cause cancer** | | | | | <.001 |
|  |  |  | Strongly agree | | 1164 (22.73) | 668 (18.52) |  |
|  |  |  | Somewhat agree | | 2308 (45.06) | 1769 (49.04) |  |
|  |  |  | Somewhat disagree | | 1023 (19.97) | 753 (20.88) |  |
|  |  |  | Strongly disagree | | 627 (12.24) | 417 (11.56) |  |
|  |  | **Cancer is unavoidable** | | | | | <.001 |
|  |  |  | Strongly agree | | 464 (9.01) | 175 (4.87) |  |
|  |  |  | Somewhat agree | | 1287 (25) | 664 (18.46) |  |
|  |  |  | Somewhat disagree | | 1938 (37.65) | 1571 (43.68) |  |
|  |  |  | Strongly disagree | | 1459 (28.34) | 1187 (33) |  |
|  |  | **Too many recommendations^i^** | | | | | <.001 |
|  |  |  | Strongly agree | | 1275 (24.63) | 727 (20.22) |  |
|  |  |  | Somewhat agree | | 2577 (49.78) | 1844 (51.29) |  |
|  |  |  | Somewhat disagree | | 887 (17.13) | 712 (19.81) |  |
|  |  |  | Strongly disagree | | 438 (8.46) | 312 (8.68) |  |
|  |  | **Obesity affects cancer onset** | | | | | <.001 |
|  |  |  | A lot | | 1540 (30.35) | 1376 (38.93) |  |
|  |  |  | A little | | 1634 (32.2) | 1295 (36.63) |  |
|  |  |  | Not at all | | 522 (10.29) | 310 (8.77) |  |
|  |  |  | Don’t know | | 1378 (27.16) | 554 (15.67) |  |
| **Numeric variables, mean (SD)** | | | | | | | |
|  | Age (years) | | | | 58.35 (0.24) | 54.41 (0.27) | <.001 |
|  | BMI | | | | 28.44 (0.09) | 28.53 (0.11) | .54 |
|  | Sitting time per day | | | | 6.58 (0.06) | 6.98 (0.06) | <.001 |

^a^Chi-square tests for categorical variables and 2-tailed *t* test for continuous variables; level of significance: *P*=.05; *P*<.05 indicates a significant difference in this variable between the use and nonuse of web-based medical records.

^b^In general, how much would you trust information about cancer from a doctor/government health agencies/charitable organizations/religious organizations and leaders? (Supplement to the variable-related questions in the survey).

^c^Not including psychiatrists and other mental health professionals, is there a particular doctor, nurse, or other health professional that you see most often? (Supplement to the variable-related questions in the survey).

^d^In the past 12 months, not counting times you went to an emergency room, how

many times did you go to a doctor, nurse, or other health professional to get care for yourself?(Supplement to the variable-related questions in the survey).

^e^Have you ever been offered online access to your medical records by your health care provider/health insurer?(Supplement to the variable-related questions in the survey).

^f^Are you currently caring for or making health care decisions for someone with a medical, behavioral, disability, or other condition?(Supplement to the variable-related questions in the survey).

^g^Over the past 2 weeks, how often have you been bothered by any of the following problems? Little interest or pleasure in doing things/Feeling down, depressed, or hopeless/Feeling nervous, anxious, or on edge.(Supplement to the variable-related questions in the survey).

^h^In the past 12 months, have you seen messages saying that a Federal Court has

ordered tobacco companies to make statements about the dangers of smoking

cigarettes? (Supplement to the variable-related questions in the survey).

^i^How much do you agree or disagree with that there are so many different recommendations about preventing cancer, it's hard to know which ones to follow?(Supplement to the variable-related questions in the survey).
